# Structures of *Streptococcus pneumoniae* PiaA and Its Complex with Ferrichrome Reveal Insights into the Substrate Binding and Release of High Affinity Iron Transporters

**DOI:** 10.1371/journal.pone.0071451

**Published:** 2013-08-12

**Authors:** Wang Cheng, Qiong Li, Yong-Liang Jiang, Cong-Zhao Zhou, Yuxing Chen

**Affiliations:** Hefei National Laboratory for Physical Sciences at the Microscale and School of Life Sciences, University of Science and Technology of China, Hefei, China; INRA Clermont-Ferrand Research Center, France

## Abstract

Iron scarcity is one of the nutrition limitations that the Gram-positive infectious pathogens *Streptococcus pneumoniae* encounter in the human host. To guarantee sufficient iron supply, the ATP binding cassette (ABC) transporter Pia is employed to uptake iron chelated by hydroxamate siderophore, via the membrane-anchored substrate-binding protein PiaA. The high affinity towards ferrichrome enables PiaA to capture iron at a very low concentration in the host. We presented here the crystal structures of PiaA in both apo and ferrichrome-complexed forms at 2.7 and 2.1 Å resolution, respectively. Similar to other class III substrate binding proteins, PiaA is composed of an N-terminal and a C-terminal domain bridged by an α-helix. At the inter-domain cleft, a molecule of ferrichrome is stabilized by a number of highly conserved residues. Upon ferrichrome binding, two highly flexible segments at the entrance of the cleft undergo significant conformational changes, indicating their contribution to the binding and/or release of ferrichrome. Superposition to the structure of *Escherichia coli* ABC transporter BtuF enabled us to define two conserved residues: Glu119 and Glu262, which were proposed to form salt bridges with two arginines of the permease subunits. Further structure-based sequence alignment revealed that the ferrichrome binding pattern is highly conserved in a series of PiaA homologs encoded by both Gram-positive and negative bacteria, which were predicted to be sensitive to albomycin, a sideromycin antibiotic derived from ferrichrome.

## Introduction

Iron is an essential component of many biological systems and plays important roles in most living organisms. Although iron is abundant in nature, free iron ion is scarce in most local environments. Under an aerobic aqueous environment at neutral pH, the concentration of free iron is only about 10^−18^ M, which is extremely low compared to the micromolar level of iron concentration required for bacteria [1]. To overcome this nutrition limitation, pathogenic and commensal bacteria have evolved alternative strategies to uptake iron from the host. In human, iron is abundant and usually exists as the protein-bound form in transferrin, ferritin, hemoglobin and cytochrome [2]. The iron in these proteins could be deprived by a wide variety of microorganisms via the low-molecular-weight (500–1000 Da) iron chelators, termed siderophores, which can bind iron with an association constant as high as 10^24^ M^−1^ [1], [3].

In Gram-negative bacteria, highly specialized siderophore-binding outer membrane receptors, such as *Escherichia coli* FepA binding to enterobactin, are first employed to transport ferric-siderophores into the periplasmic space driven by the energy-transducing TonB-ExbB-ExbD system [4], [5], [6]. Afterwards, the ferric-siderophores are forwarded to the cytosol via the inner membrane by the ATP binding cassette (ABC) transporters. In contrast, the Gram-positive bacteria can directly uptake ferric-siderophores from the environment by using the substrate-binding proteins of ABC transporters.

According to the iron-coordination groups, ferric-siderophores have been divided into three major classes: catecholates (e.g. enterobactin), hydroxamates (e.g. ferrichrome) and α-hydroxy-carboxylates (e.g. staphyloferrin A) [7], [8]. Members in one class could be recognized by the same substrate binding protein in some cases; for instance, *E. coli* FhuD can recognize a variety of hydroxamates siderophores such as ferrichrome, coprogen, desferal [9], [10].

The cyclic hexapeptide (Gly)_3_-(N-d-acetyl-N-d-hydroxy-L-ornithine)_3_, a well-defined fungal ferrichrome, could be synthesized by a group of human parasitic fungi, such as *Aspergillus fumigatus, Coccidioides immitis* and *Histoplasma capsulate* [11], [12]. Previous reports indicated that the ferrichrome secreted from fungi might be shared by a variety of bacteria such as *E. coli*, *Staphylococcus aureus* and *Streptococcus pneumoniae* [13], [14], [15].

During the invasion to human host, *S. pneumoniae* has to escape from the host immune response system, and acquire sufficient nutrients to survive and proliferate. The iron scarcity is one of the key nutrient limitations encountered by the bacteria in the human host. To obtain sufficient iron, *S. pneumoniae* evolved two major highly conserved iron ABC transporters, which are termed Piu (for pneumococcal iron uptake) and Pia (for pneumococcal iron acquisition) systems, respectively [16], [17], [18]. Pia system is responsible for the transport of hydroxamate siderophores such as ferrichrome and ferrioxamin B, whereas Piu system for the transport of heme from hemoglobin [15], [19]. Similar to *E. coli* FhuD, PiaA was demonstrated to bind the antibiotic albomycin (a derivate of ferrichrome). In addition, PiaA can bind to another antibiotic salmycin, a derivate of ferrioxamine B [15].

Compared to the traditional antibiotics, such as gentamicin and amoxicillin, albomycin is a more effective antibiotic against *S. pneumoniae* [20]. However, the arising of drug-resistant bacteria makes it an extreme emergence to develop novel antibiotics. For instance, conjugates of between siderophores and antibiotics have been applied to arrest the growth of certain bacteria [21]. Despite the structure of *E. coli* FhuD in complex with gallichrome was determined [10], the structural basis of the siderophore-binding and transportation remains largely unknown.

To gain more insights into the the molecular details of ferrichrome binding to PiaA, we solved the crystal structures of the periplasmic portion of PiaA before and after binding to ferrichrome at 2.7 Å and 2.1 Å, respectively. Although PiaA shares an overall structure similar to *E. coli* FhuD, it adopts a quite different ferrichrome-binding cleft, which enables PiaA to capture ferrichrome directly from the environment. We found that, two highly flexible segments at the entrance of the cleft adopt variable conformations, indicating their contributions to the binding and/or release of ferrichrome. Moreover, structure-based multiple-sequence alignment indicated that a series of PiaA homologs have the capacity of binding to ferrichrome, indicating the corresponding bacteria might be senstitive to albomycin.

## Materials and Methods

### Cloning, Expression and Purification of PiaA

Recombinant PiaA/Sp_1032 (to express residues Asn23-Lys341) was produced by cloning *piaA* from the genomic DNA of *S. pneumoniae* TIGR4 into a pET28a-derived expression vector with an N-terminal 6×His-tag. The N-terminal secretion signal and lipidation site were deleted from the recombinant protein. The construct was transformed into *E. coli* strain BL21-RIL (DE3) (Novagen), growing at 37°C in 2×YT culture medium (5 g of NaCl, 16 g of Bacto-Tryptone, and 10 g of yeast extract per liter) containing 30 µg/ml kanamycin and 34 µg/ml chloramphenicol. When the OD_600 nm_ reached about 1.0, the culture temperature was shifted to 16°C, and protein expression was induced with 0.2 mM isopropyl β-D-1-thiogalactopyranoside for an additional 20 hr. Cells were collected and resuspended in 40 ml lysis buffer (20 mM Tris-Cl, pH 7.5, 100 mM NaCl). After sonication for 20 min followed by centrifugation at 12,000×g for 30 min, the supernatant containing the soluble protein was collected and loaded onto a Ni-NTA column (GE healthcare) equilibrated with the binding buffer (20 mM Tris-Cl, pH 7.5, 100 mM NaCl). The target protein was eluted with 400 mM imidazole, and further loaded onto a Superdex 75 column (GE Healthcare) pre-equilibrated with 50 mM NaAc, pH 5.2. Fractions containing the target protein were pooled and concentrated to 10 mg/ml for crystallization.

The selenium-methionine (Se-Met)-labeled PiaA protein was expressed in *E. coli* strain B834 (DE3) (Novagen). Transformed cells were grown at 37°C in Se-Met medium (M9 medium with 25 µg/ml Se-Met and the other essential amino acids at 50 µg/ml) containing 30 µg/ml kanamycin until the OD_600 nm_ reached about 1.0 and were then induced with 0.2 mM isopropyl β-D-1-thiogalactopyranoside for another 20 hr at 16°C. Se-Met substituted His_6_-PiaA was purified in the same manner as described above for the native His_6_-PiaA.

### Crystallization, Data Collection and Processing

The ferrichrome (iron free) was purchased from Sigma, and its ferric form was made by mixing with FeCl_3_ at 1∶1 molar ratio. For the crystal of PiaA complex with ferrichrome, the ligand was added to 10 mg/ml PiaA at a 1∶1 molar ratio in 50 mM NaAc, pH 5.2. Both crystals of Se-Met substituted and ferrichrome-binding PiaA were grown at 289 K using the hanging drop vapor-diffusion methods, with the initial condition of mixing 1 µl protein solution with an equal volume of the reservoir solution (30% polyethylene glycol 400, 0.1 M NaAc, pH 4.6, 0.1 M CdCl_2_). The crystals were transferred to cryoprotectant (reservoir solution supplemented with 25% glycerol) and flash-cooled with liquid nitrogen. The Se-Met derivative data for a single crystal were collected at 100 K in a liquid nitrogen stream using the beamline at the Shanghai Synchrotron Radiation Facility (SSRF). The datasets were integrated and scaled with the program HKL2000 [22]. The subsequent processing of the Se-Met substituted data by PHENIX showed a severe pseudo-translation [23]. Thus another crystal obtained at 289 K with the initial condition of mixing 1 µl protein solution with an equal volume of the reservoir solution (2.4 M sodium malonate, 0.1 M Bis-tris propane, pH 7.0) was used to solve the phase problem using heavy atom methods. The crystal with iodine was obtained by quick cryo-soaking in the solution containing 300 mM KI for about 30 sec, and mounted in rayon loop for data collection.

### Structure Solution and Refinement

The structure of PiaA was determined using the single-wavelength anomalous dispersion phasing method [24] with the iodine anomalous signal using the program phenix.solve implemented in PHENIX [25]. The initial model was built automatically with the program AutoBuild in PHENIX [25]. The resultant model was subsequently used as a search model against the 2.0 Å data of PiaA in complex with Bis-tris propane. Using the PiaA structures as the search model, the structure of Se-Met substitued and ferrichrome-binding PiaA were determined by molecular replacement with the program MOLREP [26] implemented in CCP4i [27]. The Se-Met substituted and ferrichrome-binding PiaA were further refined by using the maximum likelihood method implemented in REFMAC5 [28] as part of CCP4 program suite and rebuilt interactively by using the σA-weighted electron density maps with coefficients mFo-DFc and mFo-DFc in the program COOT [29]. The structure of PiaA in complex with Bis-tris propane was further refined with the refinement program from PHENIX and rebuilt in COOT. The final models were evaluated with the programs MOLPROBITY [30] and PROCHECK [31]. The data collection, processing and structure refinement statistics were listed in Table 1. All structure figures were prepared with the program PyMol [32].

**Table 1:** Crystal parameters, data collection and structure refinement.

	PiaA-KI	PiaA-B3P	PiaA-ferrichrome	apo-PiaA
*Data collection*				
Space group	*P*2_1_2_1_2_1_	*P*2_1_2_1_2_1_	*P*2_1_2_1_2_1_	*P*2_1_2_1_2_1_
Unit cell (Å), (°)	43.52, 63.79, 136.11	43.63, 63.44, 135.80	76.10, 84.27, 93.78	76.04, 86.30, 93.53
Resolution range (Å)	50.00–2.35	50.00–2.00	50.00–2.10	50.00–2.70
Unique reflections	16,368 (813 )	26,290 (1,281)	34,376 (1,693)	17,106 (826)
Completeness (%)	100.0 (100.0)	99.9 (100.0)	96.6 (97.7)	97.8 (97.2)
<I/σ(I)>	19.7 (4.7)	21.9 (4.0)	11.2 (5.3)	11.1 (4.2)
Rmerge^a^ (%)	13.4 (67.3)	8.1 (55.3)	9.4 (21.2)	9.3 (24.5)
Average redundancy	13.6 (12.3)	7.1 (7.1)	2.9 (2.9)	3.2 (3.3)
*Structure refinement*				
Resolution range (Å)		46.35–2.00	36.86–2.10	41.12–2.70
R-factor^b^/R-free^c^ (%)		19.46/23.25	19.96/24.80	25.35/29.75
Number of protein atoms		2399	4775	4441
Number of water atoms		113	448	79
RMSD^d^ bond lengths (Å)		0.007	0.004	0.008
RMSD bond angles (°)		0.966	0.850	0.866
Mean B factors (Å^2^)		34.04	14.00	27.79
*Ramachandran plot* e *(residues, %)*				
Most favored		98.37	98.01	96.82
Additionally allowed		1.63	1.99	3.18
PDB entry		4HMO	4HMQ	4HMP

a R_merge_ = ∑_hkl_∑_i_|I_i_(hkl)- <I(hkl)>|/∑_hkl_∑_i_I_i_(hkl), where I_i_(hkl) is the intensity of an observation and <I(hkl)> is the mean value for its unique reflection; Summations are over all reflections.

b R-factor = ∑_h_|Fo(h)-Fc(h)|/∑_h_Fo(h), where Fo and Fc are the observed and calculated structure-factor amplitudes, respectively.

c R-free was calculated with 5% of the data excluded from the refinement.

d Root-mean square-deviation from ideal values.

e Categories were defined by Molprobity.

### Isothermal Titration Calorimetry Assays

Microcalorimetric titrations were carried out at 25°C employing a MicroCal ITC 200 instrument (GE Healthcare). Both samples of protein and ferrichrome were dissolved in the buffer of 50 mM sodium acetate pH 5.2, then vacuum degassed before use and injections carried out at 2-min intervals. The heat of the dilutions was determined by carrying out suitable reference titrations. The titration data were analyzed using a single-site model and evaluated with the Origin software provided by MicroCal. The affinity of PiaA and the W63A mutant towards ferrichrome is expressed as the dissociation constant (Kd).

## Results and Discussion

### Binding Affinity of PiaA Towards Ferrichrome

**Figure 1:**
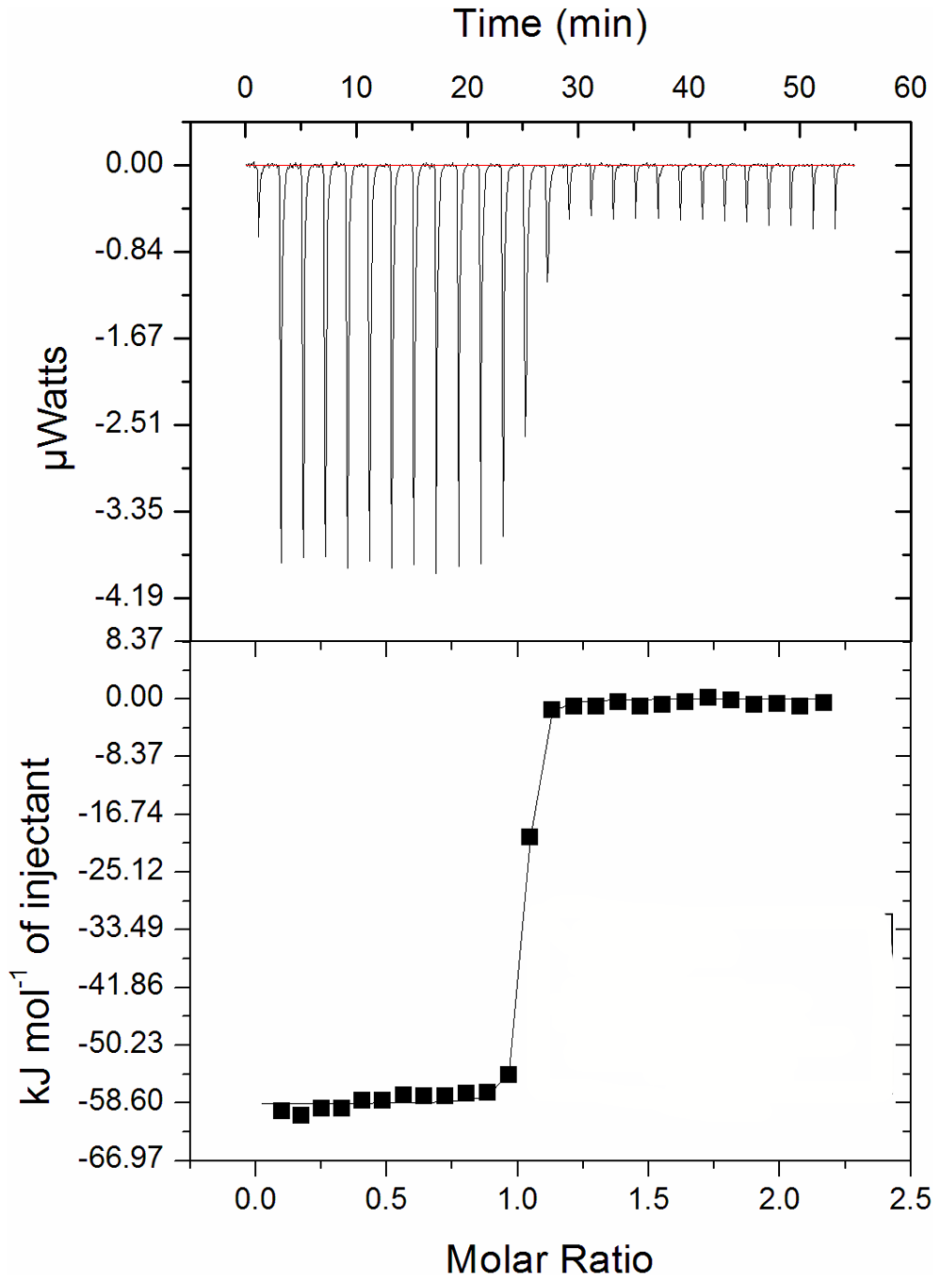
Representative raw and fit ITC isotherms for ferrichrome titrated into PiaA. Calorimetric titrations were performed at 25°C by stepwise adding 19 drops of 2 µl ferrichrome at 300 µM dissolved in 50 mM sodium acetate, pH 5.2 to 200 µl PiaA at 30 µM.

A previous report has shown that the Pia ABC transporter is responsible for the acquisition of hydroxamate siderophores in *S. pneumoniae* [15]. To determine the affinity of substrate-binding protein PiaA towards ferrichrome, the changes of heat were plotted against the molar ratio of ferrichrome to PiaA (Fig. 1). The calculated dissociation constant (Kd) of PiaA towards ferrichrome is 5.8±1.4 nM, which is about 1/20 to that of *E. coli* FhuD towards ferrichrome (Kd = 0.1 µM) [33]. This much higher affinity enables the Gram-positive bacteria *S. pneumoniae* to bind the substrates at a rather lower concentration in the environment, whereas FhuD does not require such a high affinity because the substrates have been readily enriched in the periplasmic space in *E. coli* [34].

### Overall Structure of PiaA

The crystal of PiaA in complex with ferrichrome was obtained by adding the ligand to the protein at a molar ratio of 1∶1. To solve the phase problem, Se-Met substituted PiaA was crystallized, but the diffraction data at the selenium edge showed the existence of pseudo-translation during crystal packing. A crystal from 2.4 M sodium malonate and 0.1 M Bis-tris propane (B3P), pH 7.0 was applied to soaking with 300 mM KI and eventually enabled us to solve the structure (termed PiaA-B3P) at 2.0 Å using the phases obtained from a SAD experiment at the iodine edge. The structures of Se-Met substituted apo-PiaA and ferrichrome-binding complex were subsequently solved at 2.7 Å and 2.1 Å, respectively, by molecular replacement.

In the structure of PiaA-B3P, a molecule of B3P with two conformations binds to a cleft of PiaA. Structural alignment of our PiaA-B3P with the recently released structure of B3P-binding PiaA (PDB: 4H59) from *S. pneumoniae Canada MDR_19A* yields a root mean square deviation (RMSD) of 0.24 Å over 268 Cα atoms. They share an almost identical overall structure and active-site, except that B3P molecules adopt different conformations.

**Figure 2:**
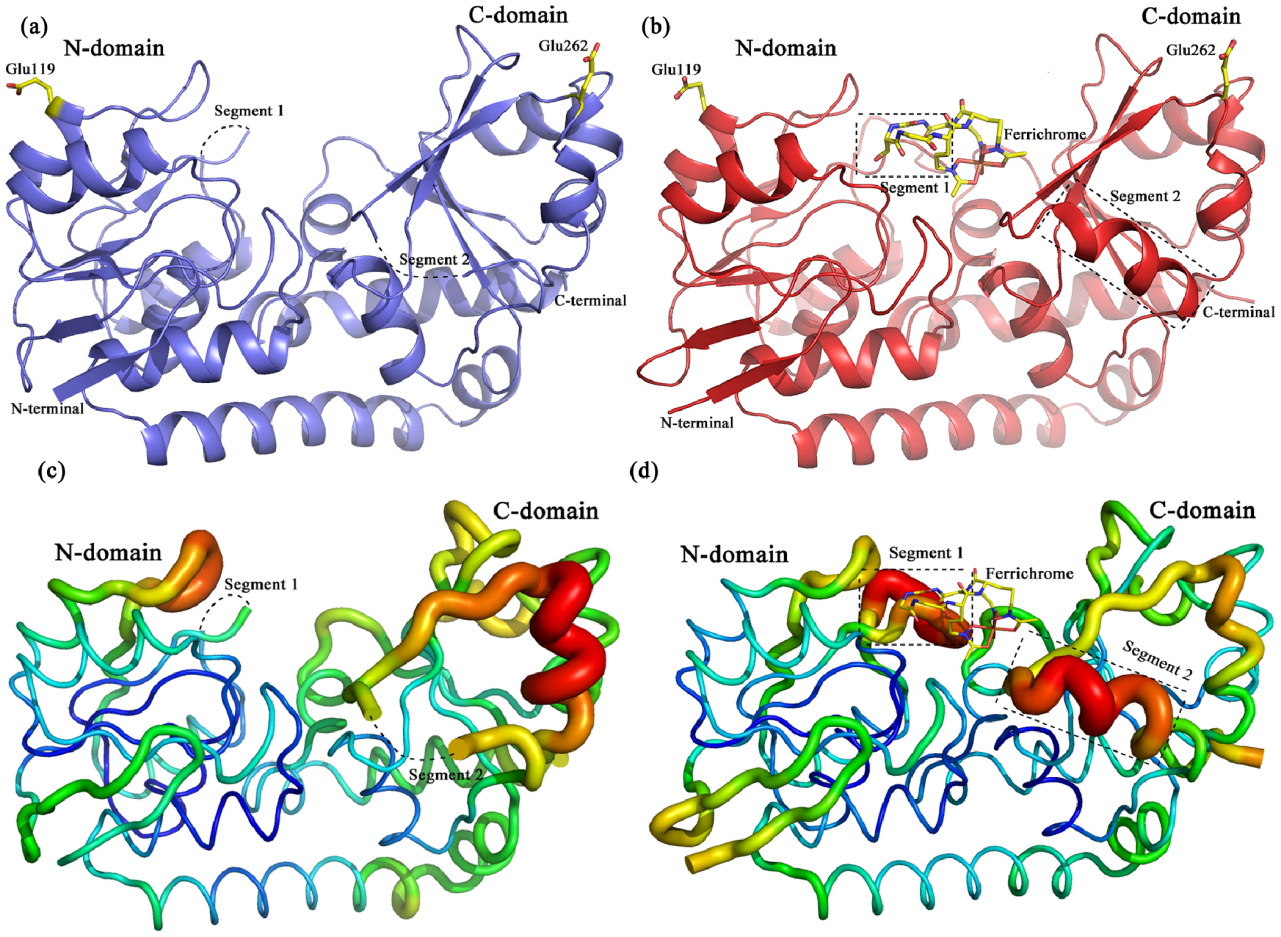
Overall structure of PiaA. **a**) apo-form. **b**) ferrichrome-complexed form. The two missing segments (residues 84–90 and 248–252) were shown in dotted lines. Ferrichrome and residues Glu119 and Glu262 were shown as sticks. B-factor tube diagrams of **c**) the apo- and **d**) complexed forms. Regions of higher B-factor are shown with larger diameter and colored in red.

Each asymmetric unit of the apo-PiaA structure contains two molecules, which are quite similar to each other with an RMSD of 0.24 Å over 278 Cα atoms. The overall structure of PiaA is composed of an individual N-terminal and a C-terminal domain linked by an α-helix (Fig. 2a). Both the N- and C-terminal domains consist of a central β-sheet sandwiched by α-helices on both sides. Similar to *E. coli* FhuD [10], this pattern of two separated domains connected by an α-helix is a common feature of the class III substrate-binding proteins [35]. The N- and C-terminal domains face each other to form a hydrophobic and plastic cleft. Segments 1 (residues Asn84−Lys90) and 2 (residues Glu248−Glu252) at the entrance of this cleft are missing from the electron density map due to their high flexibility (Fig. 2a and Fig. S1a). Notably, Segments 1 and 2 are located at the N- and C-terminal domains, respectively, indicating the cooperativity between the two domains. Different from *E. coli* FhuD, the most N-terminus of apo-PiaA structure has an extended β-hairpin, which may function as a hinge to connect the N-terminal transmembrane α-helix that is inserted in the membrane of Gram-positive bacteria.

### Conformational Changes upon Ferrichrome Binding

In the complex structure, a molecule of ferrichrome is bound at the center of the inter-domain cleft, with the iron moiety sitting against the bottom of the cleft and the backbone pointing outwards (Fig. 2b). Similar to most class III substrate-binding proteins with a relative rigid linker of α-helix, binding of ferrichrome does not trigger significant conformational changes. Superposition of apo-PiaA and PiaA-B3P against the ferrichrome-complexed form gives an RMSD of 0.31 Å for 262 Cα atoms and 0.45 Å for 283 Cα atoms, respectively.

**Figure 3:**
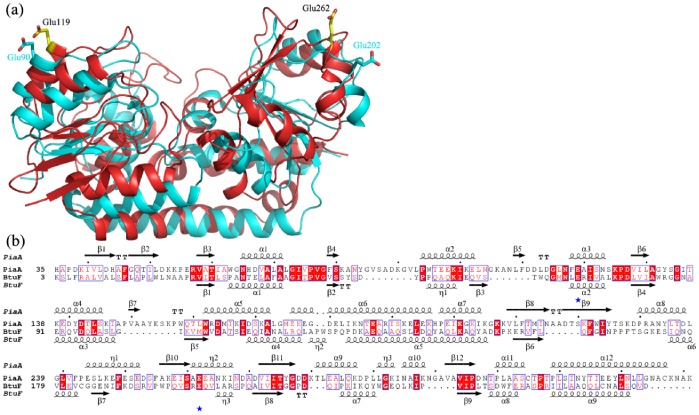
Structural comparison of PiaA and *E. coli* BtuF. **a**) Superposition of ferrichrome-complexed PiaA against *E.coli* BtuF (PDB: 2QI9). BtuF was colored in cyan. The two pairs of glutamates of PiaA and BtuF were labeled and shown as yellow and cyan sticks, respectively. **b**) Sequence alignment of PiaA and *E. coli* BtuF. The two conserved glutamates were marked with blue asterisks.

As a class III binding protein, this phenomenon of subtle conformational changes upon ligand binding has been a long-standing puzzle: how the transmembrane subunits discriminate the apo from the ligand-bound state. Recently, the complex structure of vitamin B12 ABC transporter (BtuCD-F) demonstrated that the substrate-binding protein BtuF binds to the transmembrane BtuC dimer via two pairs of salt bridge between Glu74/Glu202 from BtuF and two Arg56 from BtuC dimer, respectively [36]. Structural comparison of the apo and holo-BtuF revealed that the segment containing Glu202 adopts a different conformation for discrimination [37]. Activity assays combined with site-directed mutagenesis of the class III substrate-binding proteins *E. coli* FecB [38], *S. aureus* FhuD2 [39] and *Bacillus subtilis* FeuA [40], indicated that the corresponding Glu-Arg salt bridges are indispensible for properly docking the substrate-binding protein to the permease subunits. Thus, even subtle conformational changes at either of the two Glu regions will be transferred to the salt bridges. Superposition of PiaA-ferrichrome against the structure of BtuF (PDB code: 2QI9) combined with the sequence alignment enabled us to find two highly conserved glutamate residues: Glu119 and Glu262, which might form salt bridges with the corresponding arginine residues of PiaB and PiaC, respectively (Fig. 3a and 3b).

Upon ferrichrome binding, the Segments 1 and 2 missing in the apo-form structure undergo significant conformational changes (Fig. 2a and 2b). As a result of induced fit, residues corresponding to the two missing segments are folded into a loop and a η-helix, respectively (Fig. 2b and Fig. S1b). However, the relatively higher B-factor values of these two segments indicated they are still somewhat flexible in the complex structure (Fig. 2c and 2d). Superposition of the two molecules in each asymmetric unit (Fig. S1c) further indicated that the flexibility of these two segments could be also seen from the relative higher RMSD values (1.41 Å for Segment 1 and 0.39 Å for Segment 2) compared to the overall RMSD of 0.24 Å. In detail, residues Ser87, Ala88 and Asp89 in Segment 1 of one molecule shift towards ferrichrome against the corresponding residues of another molecule in the asymmetric unit at a distance of about 3 to 4 Å. All together, we propose that these two flexible segments, in addition to residues Glu119 and Glu262, contribute to the majority of a tunable interface with the permease subunits PiaB and PiaC.

### The Ferrichrome-binding Site

**Figure 4:**
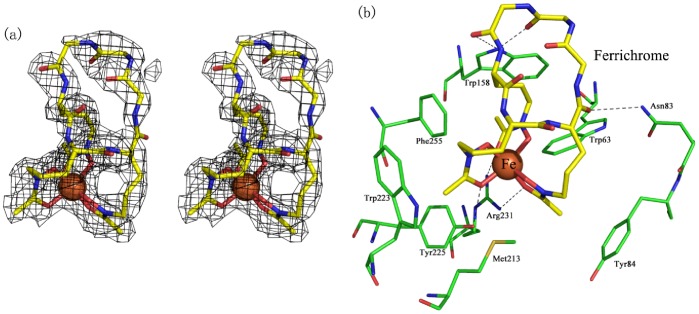
The ferrichrome binding site. **a**) The conformation of ferrichrome in the complex structure was shown as the stereo view. The omit maps were counted at 1.0 σ. **b**) The ferrichrome binding site. Ferrichrome-binding residues were labeled, and the hydrogen bonds were shown in dashed lines.

As shown in the electron density map of the complex structure, the inter-domain cleft captures a molecule of ferrichrome with Λ-cis configuration (Fig. 4a). The ferrichrome is stabilized by a couple of residues via both hydrophilic and hydrophobic interactions (Fig. 4b). The iron moiety of ferrichrome is pulled to the C-terminal domain via three hydrogen bonds with residues Arg231 and Tyr225. The two side-chain amino groups of Arg231 form two hydrogen bonds with carbonyl oxygen atoms of two hydroxamic acid moieties, respectively, whereas the hydroxyl group of Tyr225 makes a hydrogen bond with the carbonyl oxygen of the third hydroxamic acid moiety. In addition, the backbone of ferrichrome is stabilized at the opening side of the inter-domain cleft via three hydrogen bonds with the side chains of Trp158 and Asn83. These six hydrogen bonds keep the ferrichrome to adopt an orientation with the iron moiety pointing inward to the binding cleft. In addition, the methylene carbon atoms of the hydroxyornithine moieties are further packed by a hydrophobic barrel (Fig. 4b). This barrel is mainly composed of four residues (Met213, Trp223, Tyr225 and Phe255) from the C-terminal domain and three residues (Trp63, Tyr84 and Trp158) from the N-terminal domain. A combination of both intensive hydrophilic and hydrophobic interactions attributes PiaA a very high affinity towards ferrichrome. Superposition of the complex against the apo-PiaA structure revealed that most residues in the binding cleft undergo subtle conformational changes, except for residues Asn83 and Tyr84 that locate at the invisible Segment 1 in the apo-form. To further validate the contributions of ferrichrome-binding residues, we mutated one of the conserved residues, Trp63, to Ala. The W63A mutant showed a Kd towards ferrichrome of about 32.8±12.1 nM (Fig. S2), which is about 6-fold to that of the wild-type.

**Figure 5:**
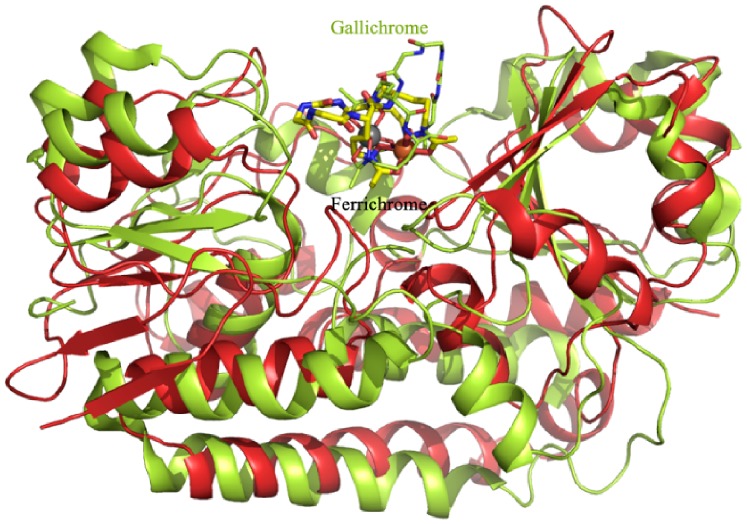
Structural comparison of PiaA and *E. coli* FhuD. FhuD and its ligand gallichrome both were colored in lemon green.

Superposition of PiaA-ferrichrome against *E. coli* gallichrome-complexed FhuD yielded an RMSD of 7.3 Å over 195 Cα atoms, with the ligands adopting different orientations (Fig. 5). Nevertheless, the ligand binding pattern of PiaA is quite similar to that of *E. coli* FhuD [10]. Although the ligands in the two structures are stabilized in a similar hydrophobic pocket by the residues from both the N- and C- domains, respectively, the metal moieties of two ligands adopt different orientations. The two C-terminal residues Arg231 and Tyr225 in PiaA to stabilize the iron moiety of ferrichrome are corresponding to the N-terminal residues Arg84 and Tyr106 of FhuD, respectively. Therefore, the metal moiety of ferrichrome is pulled by the C-terminal domain of PiaA, whereas that of gallichrome points towards the N-terminal domain of FhuD.

### A Universal Pattern to Recognize Ferrichrome by PiaA and Homologs

**Figure 6:**
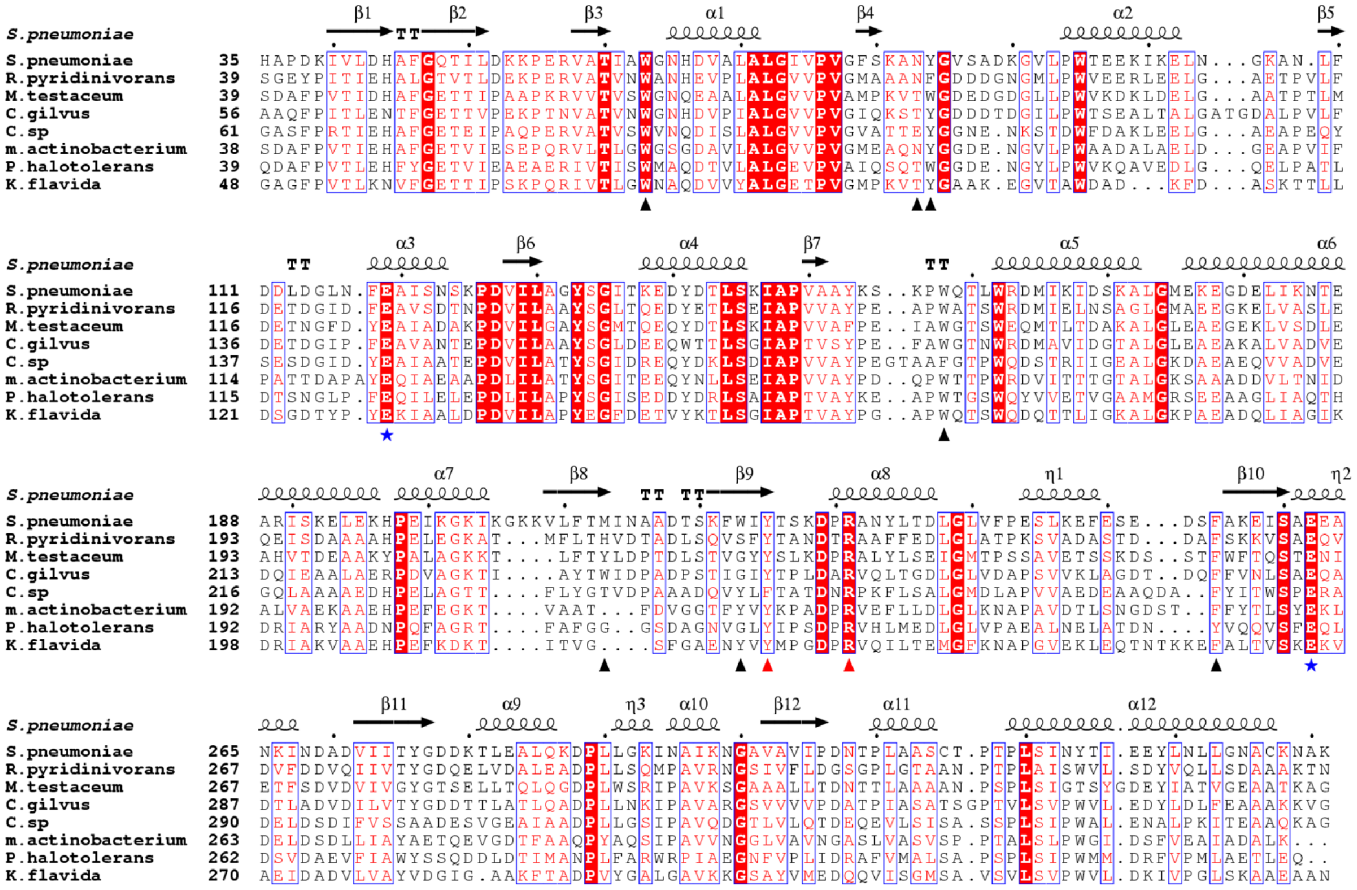
Multiple-sequence alignment of PiaA and homologs. The multiple-sequence alignment was performed with the programs Multialign [41] and Espript [42]. The secondary-structure elements of PiaA were displayed above the sequences. Residues forming hydrogen bonds with the three carbonyl oxygen atoms of the hydroxamic acid moieties were marked by red triangles. Residues participating in hydrophobic interactions with ferrichrome and hydrogen-bond with the backbone of the siderophore were labeled with black triangles. Glu119 and Glu262 were marked with blue asterisks. All sequences were downloaded from the NCBI database (www.ncbi.nlm.nih.gov). The sequences (NCBI accession numbers codes in parantheses) are *S. pneumoniae* PiaA (NP_345507.1), *Rhodococcus pyridinivorans AK37* iron-siderophore ABC transporter substrate-binding protein (ZP_09309942.1), *Microbacterium testaceum StLB037* iron hydroxamate ABC transporter periplasmic protein (YP_004226481.1), *Cellvibrio gilvus ATCC 13127* periplasmic binding protein (YP_004599979.1), *Citricoccus sp. CH26A* hypothetical protein CCH26_07037 (ZP_09825038.1), *marine actinobacterium PHSC20C1* substrate binding protein (ZP_01129479.1), *Pelagibacterium halotolerans B2* periplasmic iron-siderophore binding protein (YP_004898951.1), *Kribbella flavida DSM 17836* periplasmic binding protein (YP_003378920.1).

Sequence homology search of PiaA against the NCBI non-redundant protein database gave 74 homologs with a sequence identity of 30% or higher from both Gram-positive and negative bacteria. Based on the phylogenetic analysis, eight representative sequences were selected for the subsequent multiple-sequence alignment (Fig. 6). Most ferrichrome-binding residues are highly conserved in all species. Except for one case in *Citricoccus sp. CH26A* with a variation of Tyr to Phe, residues Arg231 and Tyr225 that forms hydrogen bonds with the iron moiety are strictly conserved, indicating the iron moiety of ferrichrome also points towards the C-terminal domain of these proteins. Other residues composed of the ferrichrome-binding cleft of PiaA, except for Met213 and Trp225, are also conserved. Taken together, these homologs should bind to the ferrichrome and its derivates in a pattern similar to that of PiaA. Moreover, all homologs employ the two highly conserved glutamate residues corresponding to Glu119 and Glu262 of PiaA to form the salt bridges. It was reported that the antibiotic albomycin, a derivate of ferrichrome, could be captured at a very low concentration via PiaA and subsequently kill *S. pneumoniae* [15]. Thus we proposed that all bacterial species possessing a PiaA homolog should be sensitive to albomycin at a certain extent.

## Supporting Information

Figure S1 **Induce fit of the two highly flexible segments.** **a**) The density map of the two missing segments as stereoviews in the apo structure. The two molecules in the asymmetry unit were separately colored in blue and orange. The terminal residues of the two missing segments were shown as yellow sticks and counted at 1.0 σ in the omit map. **b**) The density map of the two segments as stereoviews in the PiaA-ferrichrome structure. The two molecules in the asymmetry unit were shown in red and green, respectively. The residues of the two segments were exhibited as yellow sticks and counted at 1.0 σ in the omit map. **c**) Superposition of the two independent molecules in PiaA-ferrichrome structure. The two segments were boxed with rectangles of dotted lines.
(TIF)

Figure S2 **Representative raw and fit ITC isotherms for ferrichrome titrated into the W63A mutant.** Calorimetric titrations were performed at 25°C by stepwise adding 19 drops of 2 µl ferrichrome at 300 µM dissolved in 50 mM sodium acetate, pH 5.2 to 200 µl PiaA W63A mutant at 30 µM.
(TIF)
