# Supplementary figures and images for: Structures of Streptococcus pneumoniae PiaA and Its Complex with Ferrichrome Reveal Insights into the Substrate Binding and Release of High Affinity Iron Transporters
Source: PLoS One. 2013 Aug 12;8(8):e71451. doi: 10.1371/journal.pone.0071451 (PMC3741162; doi:10.1371/journal.pone.0071451)

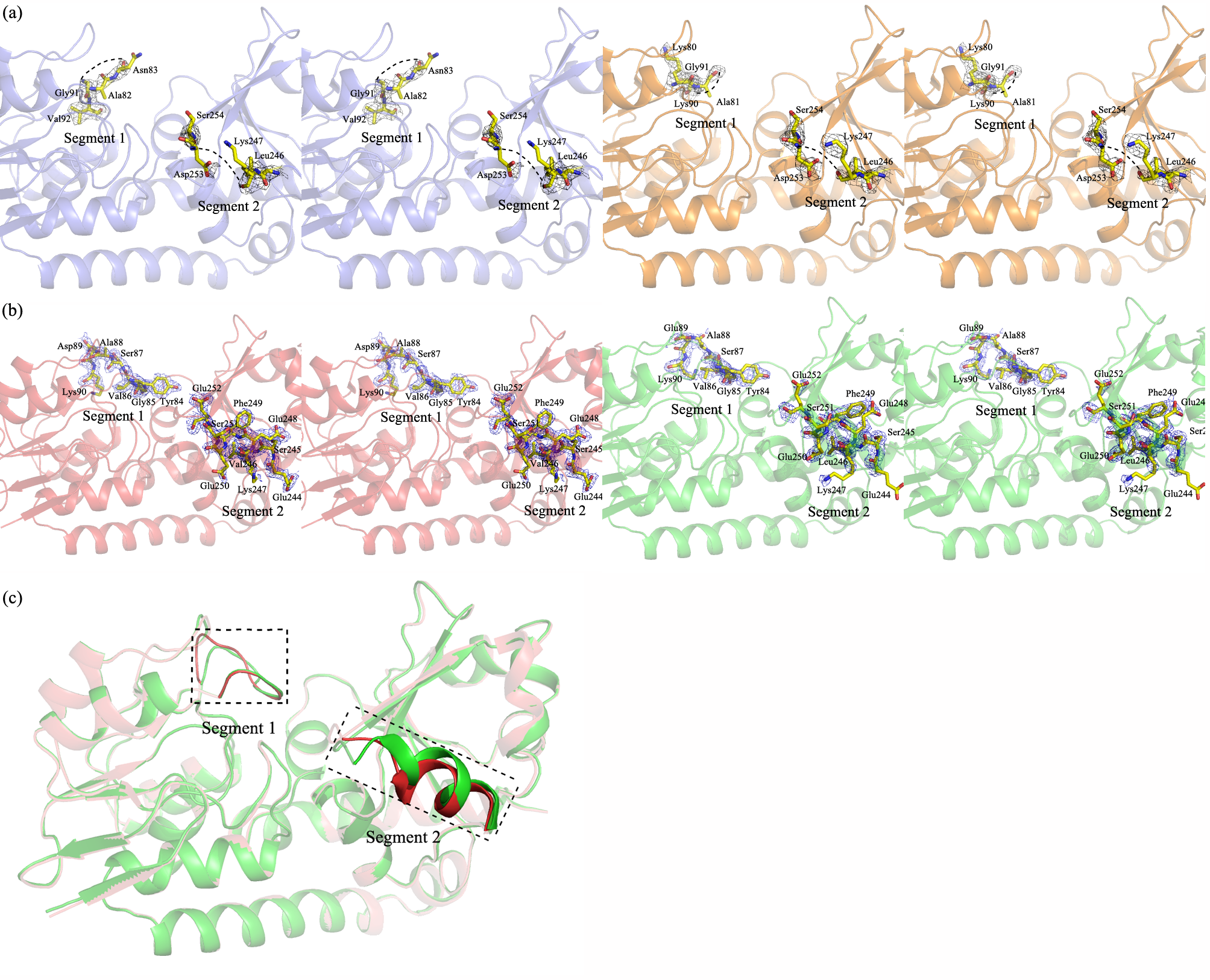

Supplement: Figure S1 — Induce fit of the two highly flexible segments. a) The density map of the two missing segments as stereoviews in the apo structure. The two molecules in the asymmetry unit were separately colored in blue and orange. The terminal residues of the two missing segments were shown as yellow sticks and counted at 1.0 σ in the omit map. b) The density map of the two segments as stereoviews in the PiaA-ferrichrome structure. The two molecules in the asymmetry unit were shown in red and green, respectively. The residues of the two segments were exhibited as yellow sticks and counted at 1.0 σ in the omit map. c) Superposition of the two independent molecules in PiaA-ferrichrome structure. The two segments were boxed with rectangles of dotted lines. (TIF) [file pone.0071451.s001.tif]

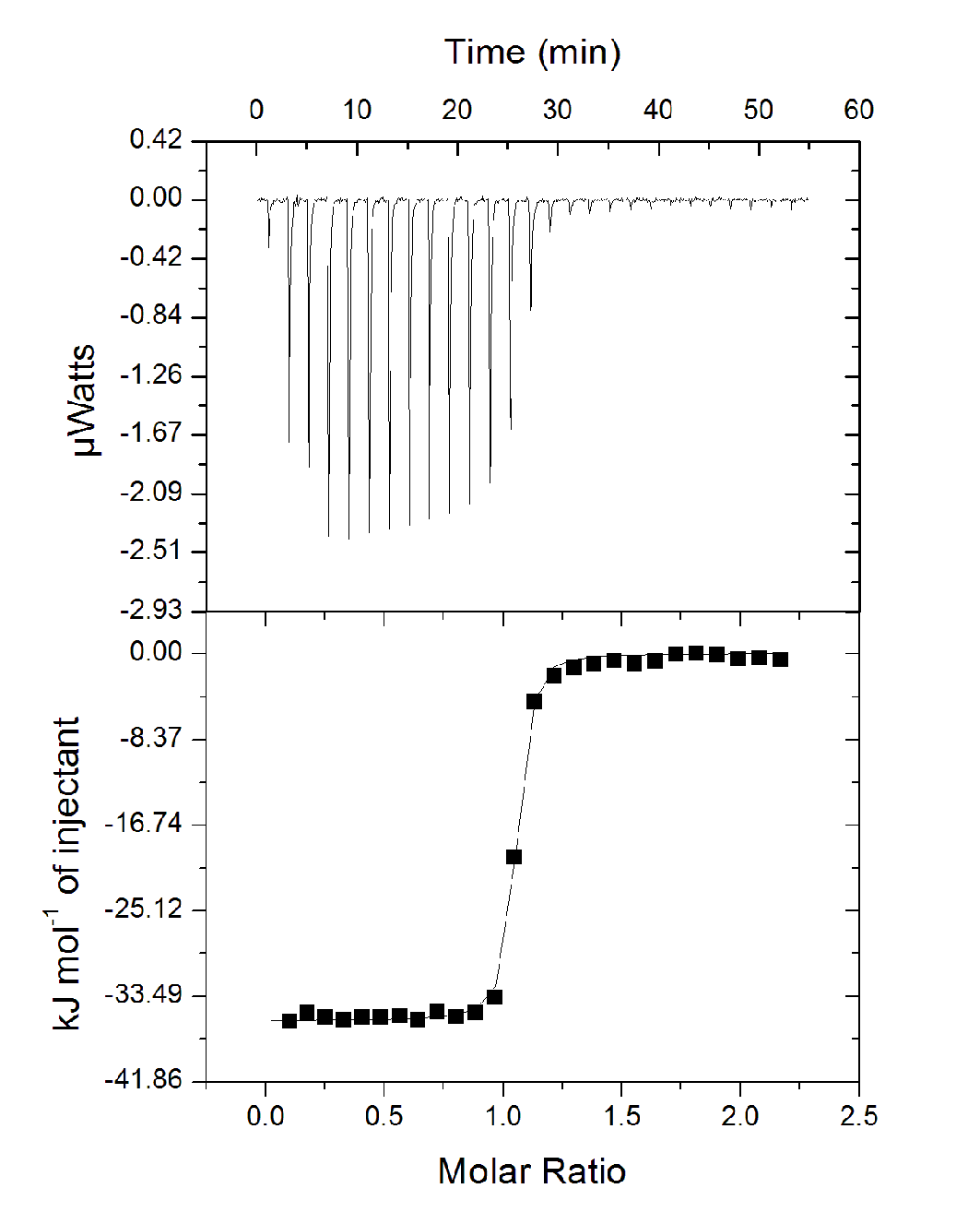

Supplement: Figure S2 — Representative raw and fit ITC isotherms for ferrichrome titrated into the W63A mutant. Calorimetric titrations were performed at 25°C by stepwise adding 19 drops of 2 µl ferrichrome at 300 µM dissolved in 50 mM sodium acetate, pH 5.2 to 200 µl PiaA W63A mutant at 30 µM. (TIF) [file pone.0071451.s002.tif]
